# Innate and Adaptive Cell-Mediated Immune Responses to a COVID-19 mRNA Vaccine in Young Children

**DOI:** 10.1093/ofid/ofad608

**Published:** 2023-12-02

**Authors:** Adriana Weinberg, Michael J Johnson, Krystle Garth, Elena W Y Hsieh, Ross Kedl, Daniela Weiskopf, Mattie Cassaday, Cody Rester, Berenice Cabrera-Martinez, Ryan M Baxter, Myron J Levin

**Affiliations:** Department of Pediatrics, University of Colorado Anschutz Medical Campus, Aurora, Colorado, USA; Department of Medicine, University of Colorado Anschutz Medical Campus, Aurora, Colorado, USA; Department of Pathology, University of Colorado Anschutz Medical Campus, Aurora, Colorado, USA; Department of Pediatrics, University of Colorado Anschutz Medical Campus, Aurora, Colorado, USA; Department of Pediatrics, University of Colorado Anschutz Medical Campus, Aurora, Colorado, USA; Department of Pediatrics, University of Colorado Anschutz Medical Campus, Aurora, Colorado, USA; Department of Microbiology and Immunology, University of Colorado Anschutz Medical Campus, Aurora, Colorado, USA; Department of Microbiology and Immunology, University of Colorado Anschutz Medical Campus, Aurora, Colorado, USA; Center for Infectious Disease and Vaccine Research, La Jolla Institute for Immunology, La Jolla, California, USA; Division of Infectious Diseases and Global Public Health, Department of Medicine, University of California, San Diego, La Jolla, California, USA; Department of Microbiology and Immunology, University of Colorado Anschutz Medical Campus, Aurora, Colorado, USA; Department of Microbiology and Immunology, University of Colorado Anschutz Medical Campus, Aurora, Colorado, USA; Department of Microbiology and Immunology, University of Colorado Anschutz Medical Campus, Aurora, Colorado, USA; Department of Microbiology and Immunology, University of Colorado Anschutz Medical Campus, Aurora, Colorado, USA; Department of Pediatrics, University of Colorado Anschutz Medical Campus, Aurora, Colorado, USA; Department of Medicine, University of Colorado Anschutz Medical Campus, Aurora, Colorado, USA

**Keywords:** cell-mediated immunity, children, COVID-19 mRNA vaccines, SARS-CoV-2 infection, trained immunity

## Abstract

**Background:**

There is little information on cell-mediated immunity (CMI) to COVID-19 mRNA vaccines in children. We studied adaptive and innate CMI in vaccinated children aged 6 to 60 months.

**Methods:**

Blood obtained from participants in a randomized placebo-controlled trial of an mRNA vaccine before and 1 month after the first dose was used for antibody measurements and CMI (flow cytometry).

**Results:**

We enrolled 29 children with a mean age of 28.5 months (SD, 15.7). Antibody studies revealed that 10 participants were infected with SARS-CoV-2 prevaccination. Ex vivo stimulation of peripheral blood mononuclear cells with SARS-CoV-2 spike peptides showed significant increases pre- to postimmunization of activated conventional CD4+ and γδ T cells, natural killer cells, monocytes, and conventional dendritic cells but not mucosa-associated innate T cells. Conventional T-cell, monocyte, and conventional dendritic cell responses in children were higher immediately after vaccination than after SARS-CoV-2 infection. The fold increase in CMI pre- to postvaccination did not differ between children previously infected with SARS-CoV-2 and those uninfected.

**Conclusions:**

Children aged 6 to 60 months who were vaccinated with a COVID-19 mRNA vaccine developed robust CMI responses, including adaptive and innate immunity.

Vaccination has been instrumental in controlling the COVID-19 pandemic. Among the many types of COVID-19 vaccines, the most commonly used in the United States have been the mRNA vaccines [1].

Protection generated by the COVID-19 vaccines involves humoral and cell-mediated immunologic memory that is typically deployed upon encounter with the virus [2]. Vaccine-induced humoral and conventional T-cell responses to SARS-CoV-2 that contribute to protection against severe infection have been extensively studied in adults and adolescents [2–4]. As compared with antibodies, cell-mediated immunity has longer persistence and better conserved functionality against SARS-CoV-2 variants [5–7]. Nevertheless, studies in young children have focused on vaccine-induced antibody responses to multiple vaccine platforms, except for a single study of cell-mediated immunologic responses to CoronaVac [8–10]. Moreover, the number of pediatric hospitalizations increased with the appearance of the Omicron variants, underscoring the importance of studying cell-mediated immunity in vaccinated children [11]. Innate immunity, which has correlated with severity of COVID-19 disease in adults, was studied only in adult recipients of vectored COVID-19 vaccines and not at all in children [12].

To fill these knowledge gaps, we measured conventional and unconventional T-cell, natural killer (NK) cell, and antigen-presenting cell (APC) responses to SARS-CoV-2 spike antigen in samples available from children who received a first dose of COVID-19 mRNA vaccine.

## METHODS

### Study Population

Between 10 November 21 and 19 January 22, we enrolled children aged 6 to 60 months in a mRNA-1273 COVID-19 vaccine (Moderna) study, which was double-blind, randomized 3:1, and placebo controlled. Children were immune competent, had no severe underlying medical conditions, and were not known to have had previous SARS-CoV-2 infection. The study was approved by the Colorado Multiple Institutional Review Board. Parents or legal guardians signed informed consent. Blood collected before vaccination and 1 month after the first dose of vaccine was used to measure antibody and cell-mediated immunity against spike SARS-CoV-2 and control antigens.

### Antibody Measurements

Antibodies in plasma were measured against the receptor-binding domain (RBD) of the spike protein and against the nucleocapsid (N) protein. A multiplex microsphere immunoassay (LEGENDplex; BioLegend) was utilized with carboxyl beads conjugated to recombinant RBD or N, produced in positive control (*Escherichia coli* or tetanus toxoid; MilliporeSigma) or negative control (bovine serum albumin; MilliporeSigma) as previously described [13]. SARS-CoV-2 infection was defined by an anti-RBD geometric mean fluorescence intensity ≥1000 and/or anti-N mean fluorescence intensity ≥3000 prior to vaccination [13]. Infection in the first 30 days after the first dose of vaccine was defined by RBD and/or N seroconversion in placebo recipients or N seroconversion in vaccine recipients.

### Flow Cytometry Assays

Peripheral blood mononuclear cells (PBMCs) were cryopreserved within 8 hours of collection as previously described [14]. Cryopreserved PBMCs were thawed and resuspended at 10^6^ cells/mL in RPMI 1640 (Corning) containing 10% fetal bovine serum (Gemini Bio-Products), 1% glutamine (Gemini Bio-Products), 1% penicillin-streptomycin (Gemini Bio-Products), and HEPES buffer (Corning). Cells were added to triplicate wells of polypropylene microtiter plates at 250 to 500 µL/well and treated with SARS-CoV-2 spike overlapping peptides at 1 µg/mL (Megapool [15]) added by anti-CD28 (340975; BD) and anti-CD49d (340976; BD) at 1 μg/mL, R848 positive control at 0.05 µg/mL (3611-5x; Mabtech), and medium negative control without additives based on optimization assays showing that the addition of anti-CD28 and anti-CD49d to medium did not increase T-cell stimulation. R848 was used as a positive control for all cell subsets, as its addition to culture medium typically increases the expression of activation markers on conventional T cells and innate immune cells.

PBMCs from a SARS-CoV-2–seropositive leukopack control were included on each plate for assay validation. After overnight incubation at 37 °C in a humidified 5% CO_2_ atmosphere, cells were stained with zombie aqua (Biolegend 423101) viability dye and monoclonal antibodies recognizing the following cell surface markers: CD19 (BV605, clone SJ25C1, 363024; BioLegend), CD20 (BV605, clone 2H7, 302334; BioLegend), CD3 (Ax700, clone UCH1, 557943; BD), CD8 (BV570, clone RPA-T8, 301038; BioLegend), TCR γδ (PE, clone 11F2, 347907; BD), TCR Va7.2 (BV785, clone 3C10, 351722; BioLegend), CD69 (PE-Cy7, clone FN50, 557745; BD), CD137 (APC-Cy7, clone 4B4-1, 309834; BioLegend), HLA-DR (BV421, clone L243, 307636; BioLegend), CD14 (BV650, clone M5E2, 301836; BioLegend), CD56 (APC, clone 5.1H11, 362504; BioLegend), CD16 (PE-CF594, clone 3G8, 562293; BD), CD123 (PerCP-Cy5.5, clone 6H6, 306016; BioLegend), CD11c (BB515, clone B-Ly6, 564490; BD), and PD-L1 (BV711, clone 29E.ZA3, 329722; BioLegend). Brilliant stain buffer (566385; BD) was included at 10 μL/well. Cells (≥250,000 events) were analyzed with the Quanteon instrument (Agilent) and FlowJo software (Becton-Dickinson). Supplementary Figure 1 shows the gating strategy.

### Statistical Analysis

Descriptive analysis was used to summarize the demographic characteristics of the study population. Immune responses between groups were compared with unpaired tests, and those between visits within the same group were compared with paired tests. Nonparametric tests were used due to the skewed distribution of the data and/or small number of samples. Analyses were performed with Prism version 10.0.1 (GraphPad).

## RESULTS

### Characteristics of the Study Population

We enrolled 29 participants: 23 vaccine and 6 placebo recipients (Table 1). The average age at enrollment was 28.5 months, 15 were male, and the majority were White non-Hispanic. Antibody results revealed that 10 participants had prior infection (Supplementary Figure 2), including 8 vaccine recipients. In addition, 1 placebo recipient had intercurrent infection.

**Table 1.** Demographic Characteristics

Characteristic	Vaccine (n = 23)	Placebo (n = 6)	All (N = 29)
Age, mo, mean (SD)	30.4 (16.6)	21.2 (9.3)	28.5 (15.7)
Sex: male	12	3	15
Race			
White	19	4	23
Asian	2	1	3
Black	1	1	2
American Indian	1	…	1
Ethnicity: non-Hispanic	19	4	23
SARS-CoV-2 infection			
Previous	8	2	10
Intercurrent	0	1	1

### Conventional T-Cell Responses to Vaccination

**Figure 1.**
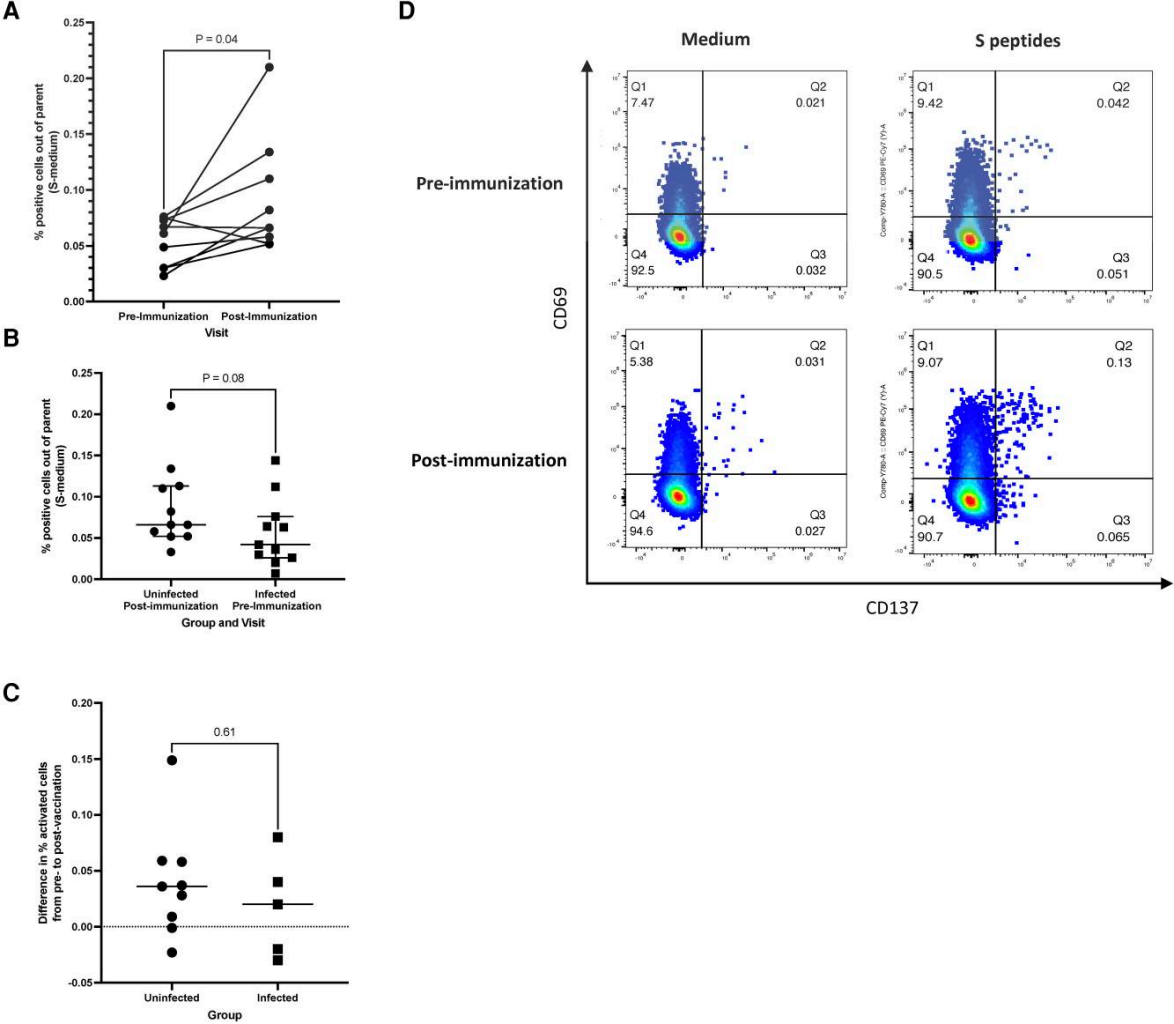
Conventional T-cell responses to the first dose of COVID-19 mRNA vaccine in children. Data were derived from 29 children. *A* and *B*, Proportions of CD4+CD69+ CD137+ T cells in spike (S) peptide–stimulated peripheral blood mononuclear cells after subtraction of unstimulated controls (S-medium) out of total CD4+ T cells. *A*, There were significantly increased proportions of spike-specific CD4+ T cells 30 days postvaccination as compared with prevaccination in children previously uninfected. *P* value was calculated by a Wilcoxon matched-pairs signed rank test. *B*, The proportions of spike-specific CD4+ T cells at 30 days postvaccination in children naive to SARS-CoV-2 tended to be higher than in children previously infected before vaccination. Horizontal line, median; error bars, IQR. *P* value was calculated by a Mann-Whitney test. *C*, Fold rises in spike-specific CD4+ T cells postvaccination as compared with prevaccination in children previously infected with SARS-CoV-2 and uninfected. Horizontal line, median. *P* value was calculated by a Mann-Whitney test. *D*, Typical representation of the CD4 T-cell responses.

CD4+ and CD8+ conventional T-cell responses to a pool of spike peptides were measured before and after the first dose of mRNA vaccine. Nine vaccine recipients without evidence of prior infection and paired samples showed significant increases in CD4+ T-cell activation by expression of CD69 and CD137 in response to spike peptide pool stimulation after subtraction of the unstimulated control (median difference, 0.04%; *P* = .04; Figure 1*A*). The proportions of activated CD4+ T cells after the first dose of vaccine in 11 children previously uninfected tended to be higher than responses to prevaccination infection in 11 children (median, 0.07% vs 0.04%), without reaching statistical significance (*P* = .08; Figure 1*B*). Among children previously infected who received active vaccine, only 5 had pre- and postvaccination samples. In these 5 children, the difference between pre- and postvaccine spike-specific CD4+ T cells did not reach statistical significance (Supplementary Figure 3). However, the increase in spike-specific conventional CD4+ T-cell activation in response to vaccination did not differ in 13 vaccinees: 5 previously infected and 8 uninfected (median increase, 0.02% and 0.04%; Figure 1*C*). CD8+ T-cell responses to the vaccine were not observed (Supplementary Figure 4).

### Innate Immune Responses to Vaccination

**Figure 2.**
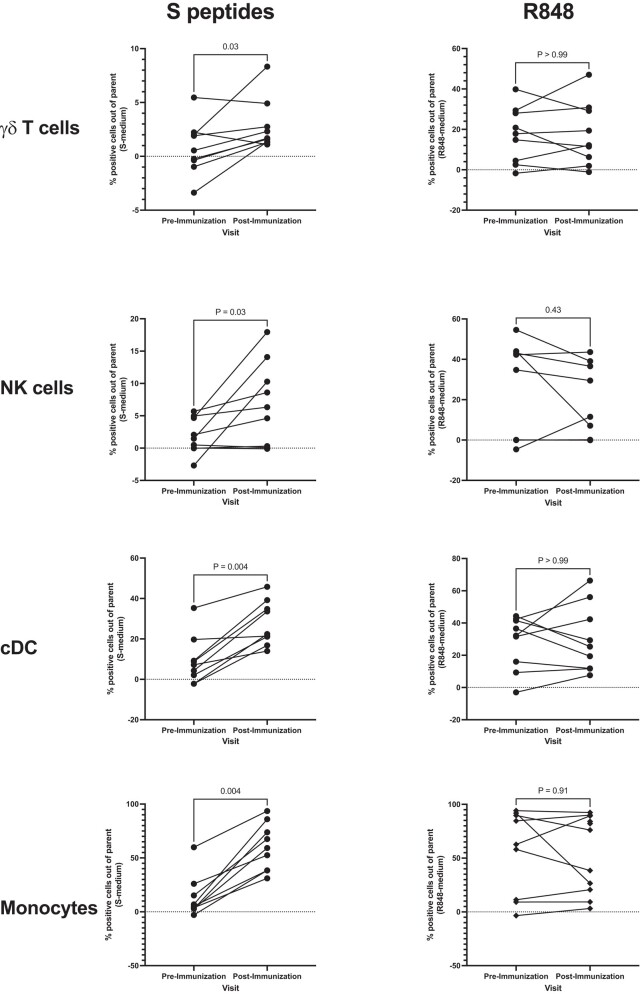
Innate immune responses to a COVID-19 mRNA vaccine in children. Data were derived from 9 children naive to SARS-CoV-2 with paired peripheral blood mononuclear cell samples before and after vaccination. Paired proportions of the activated innate immune cells are indicated before and after stimulation. *Left*, Spike (S) peptide–stimulated peripheral blood mononuclear cells after subtraction of unstimulated controls (S-medium). *Right*, R848-stimulated conditions after subtraction of unstimulated controls (R848 medium). Activated natural killer (NK) and γδ T cells were identified by the expression of CD69 and CD137 markers and expressed out of total NK and γδ T cells. Activated monocytes and conventional dendritic cells (cDCs) were identified by the expression of PDL-1 and expressed as proportions of the total monocytes and cDCs. *P* values were calculated with a Wilcoxon matched-pairs signed rank test.

We measured the responses of conventional dendritic cells (cDCs), plasmacytoid dendritic cells, monocytes, NK cells, mucosa-associated innate T cells (MAITs), and γδ T cells to ex vivo stimulation with spike peptides or R848 Toll-like receptor (TLR) agonist and unstimulated control. Activation was identified by the expression of (1) CD69 and/or CD137 on stimulated NK or T cells after subtraction of the unstimulated control and (2) PDL-1 on stimulated APCs after subtraction of the unstimulated control. In vaccine recipients who were uninfected, γδ T cells, NK cells, cDCs, and monocytes showed significantly increased net activation in response to spike peptides pre- to postvaccination (Figure 2). A correlation analysis between the proportion of innate and CD4+ conventional T cells activated by the spike peptides ex vivo postimmunization did not reveal significant associations (ρ = −0.08 to 0.49, *P* = .12–.81; not depicted). There were no significant changes in the responses to R848 stimulation pre- to postvaccination in any of the innate cell subsets.

**Figure 3.**
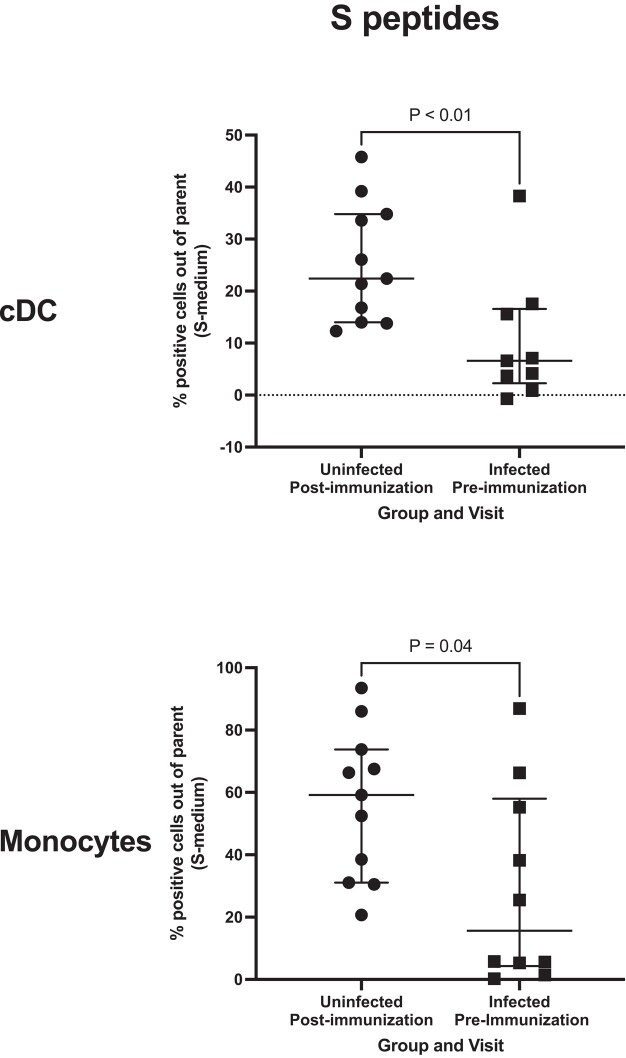
Innate immune responses to COVID-19 mRNA vaccine and SARS-CoV-2 infection in children. Data were derived from 29 children. Proportions of PDL-1+ conventional dendritic cells (cDCs) and monocytes in spike-stimulated conditions after subtraction of unstimulated conditions (S-medium). Parent populations were total cDCs and monocytes. Horizontal line, median; error bars, IQR. *P* values were calculated by a Mann-Whitney test.

Innate immune cell activation in response to spike peptide pool stimulation in vaccine recipients who were previously uninfected showed higher frequencies of activated cDCs and monocytes than observed in children infected prior to immunization (Figure 3). Nonspecific responses of innate immune cells to ex vivo R848 stimulation were similar in the 2 groups (not depicted).

The comparison of spike-induced activation pre- to postvaccination in 5 children previously infected and paired PBMC samples did not show significant increases (not depicted). However, the comparison of the spike-induced change in conventional T-cell and innate immune cell activation pre- to postimmunization in children previously infected and uninfected was also not appreciably different (not depicted).

## DISCUSSION

We describe conventional cell-mediated immunologic responses to a COVID-19 mRNA vaccine in children aged 6 to 60 months. As in older children and adults, the vaccine induced significant CD4+ T-cell responses. The magnitude of the memory response to a single dose of vaccine was similar to that after natural infection. However, responses to the vaccine were studied soon after immunization, and the timing with respect to the SARS-CoV-2 infection was not known such that the responses in children who were infected may have already declined from peak to steady state. The effect of previous infection on the conventional T-cell responses to the vaccine was not clear. In children previously uninfected, it was clear that vaccination induced a CD4+ T-cell response, but in those previously infected we were unable to show a significant increase. Yet, this finding may have resulted from the small sample size, although studies in adults found variable results when comparing adaptive cell-mediated immunologic responses in vaccinees previously infected and uninfected [16].

Unconventional T-cell and other innate cell-mediated immunologic responses to COVID-19 vaccines have been insufficiently described in any population [12]. To fill this gap, we evaluated NK, MAIT, γδ T-cell, monocyte, plasmacytoid dendritic cell, and cDC responses in our study population. The mRNA vaccine significantly increased spike-stimulated NK, γδ T-cell, monocyte, and cDC responses ex vivo in these young children. This observation is important because innate immune cells can be rapidly deployed at the entry portal for microorganisms and thereby eliminate or decrease the load of invading pathogens. In addition, innate cells critically contribute to the development of antigen-specific adaptive immunity and establish feedback mechanisms that allow adaptive immune cells to boost the innate immune cells [17–23]. Recent evidence revealed that innate immune cells develop epigenetic modifications in response to vaccines that increase their intrinsic ability to respond to antigenic challenges [24]. Epigenetic modifications and/or clonal expansion may become fixed and confer to innate immune cells memory-like responses known as *trained immunity* [19, 23, 25–35]. Innate immune responses to vaccines were studied in the context of bacille Calmette-Guerin, adjuvanted H5N1 influenza, and recombinant zoster vaccines and vectored simian immunodeficiency virus, Ebola, and COVID-19 vaccines—all of which generated trained immunity [30, 36–41]. Our observations are consistent with the development of trained immunity after the administration of mRNA COVID-19 vaccine to children.

Importantly, while we showed the development of trained immunity in response to vaccine-homologous peptide stimulation, we could not demonstrate enhanced responses to R848, which we used as a heterologous control. This observation is not novel. Development of exclusively homologous trained immunity has been well demonstrated for NK and γδ T cells in the context of cytomegalovirus (CMV) infection [42, 43]. In fact, CMV-specific NK cells show decreased activity against heterologous stimulants [44]. Less is known about exclusively homologous trained immunity in monocytes and cDCs. R848 binds to TLRs 7 and 8, eliciting downstream activation of interferon regulatory factors and NFκB [45, 46]. In contrast, peptide binding to major histocompatibility complex (MHC) class II activates Src kinases and subsequent tyrosine phosphorylation as the main intracellular downstream signaling mechanism [47]. Moreover, T-cell recognition of the peptides presented in the context of MHC class II on APCs creates an immunologic synapse where CD80, CD86, and CD40 APC receptors bind to their T-cell ligands, triggering additional activation signals in the APCs [48, 49] very distinct from those generated by R848 binding to TLRs. We propose that epigenetic modifications in the downstream signaling pathways triggered by peptide binding to MHC class II and/or costimulatory receptors on APCs accounts for the development of homologous trained immunity after administration of COVID-19 mRNA vaccines. Additional studies are needed to test this hypothesis.

Among the innate immune responses that we investigated, the activation of cDCs and monocytes by spike peptides was higher in children immunized with a single dose of vaccine than in children with prior SARS-CoV-2 infection. Additional studies are needed to compare the durability of trained immunity after vaccination and SARS-CoV-2 infection. Factors such as the adjuvant effect of the lipid encapsulation of the mRNA vaccine and differences in antigen presentation and site of inoculation between the vaccine and SARS-CoV-2 infection may contribute to the difference in the innate immune responses described after vaccination or infection. The persistent and potential protective role of the COVID-19 mRNA vaccine–generated trained immunity against SARS-CoV-2 infection warrants additional investigation, since deficient innate immunity plays a prominent role in the pathogenesis of COVID-19 [50].

We used nonspecific stimulation with R848, which is a potent agonist of TLRs 7 and 8, to investigate the development of heterologous trained immunity. Many times, the epigenetic modifications induced by vaccines confer homologous and heterologous trained immunity [35]. However, here we did not find heterologous trained immunity. To allow the detection of heterologous trained immunity, the concentration of R848 in our assays was carefully titrated to target the dynamic range of the R848 response curve rather than maximum stimulation. Hence, the discrepancy of the responses to R848 and spike could not be explained by the experimental conditions. The discrepancy was also unlikely to result from a bystander effect triggered by cytokines secreted by activated conventional CD4+ T cells. We addressed this by showing the lack of correlation between CD4+ T-cell and innate cell responses to vaccination. In addition, MAITs and conventional CD8+ T cells, which most commonly participate in bystander activation [51, 52], did not increase postvaccination as compared with prevaccination. There are previous examples where innate immune cells acquired epigenetic modifications that conferred homologous but not heterologous trained immunity. The best examples are CMV- and tumor-specific NK or γδ T cells, which display only increased homologous cytotoxic responses [42, 44]. In fact, CMV-specific NK cells have decreased responses to other pathogens [44]. Additional studies are needed to investigate trained immunity generated by COVID-19 mRNA vaccines in adults and the epigenetic modifications underlying these responses.

Our study had limitations resulting from the small number of participants with pre- and postimmunization samples, particularly in the previously infected group, and from the limited number of cells obtained from each participant. In addition, we did not have access to blood collection after the second dose of vaccine.

In conclusion, this study showed that innate and adaptive cell-mediated immunologic responses developed after COVID-19 mRNA vaccine administration in young children. The role of the diverse innate immune responses in protection against SARS-CoV-2 infection warrants further investigation.

## Supplementary Material

ofad608_Supplementary_Data

## Supplementary Data

Supplementary materials are available at *Open Forum Infectious Diseases* online. Consisting of data provided by the authors to benefit the reader, the posted materials are not copyedited and are the sole responsibility of the authors, so questions or comments should be addressed to the corresponding author.

## Notes

***Financial support*.** This work was supported by the National Institutes of Health (grants 1R01 HD107793-01A1, R01 AI154624, R01 HD102299, and 1U01 AI141919-01 to A. W.; contract 75N93019C0t0065 to D. W.).
