# Supplementary material for: Innate and Adaptive Cell-Mediated Immune Responses to a COVID-19 mRNA Vaccine in Young Children
Source: Open Forum Infect Dis. 2023 Dec 2;10(12):ofad608. doi: 10.1093/ofid/ofad608 (PMC10721446; doi:10.1093/ofid/ofad608)
Supplement: ofad608_Supplementary_Data [file ofad608_supplementary_data.docx]

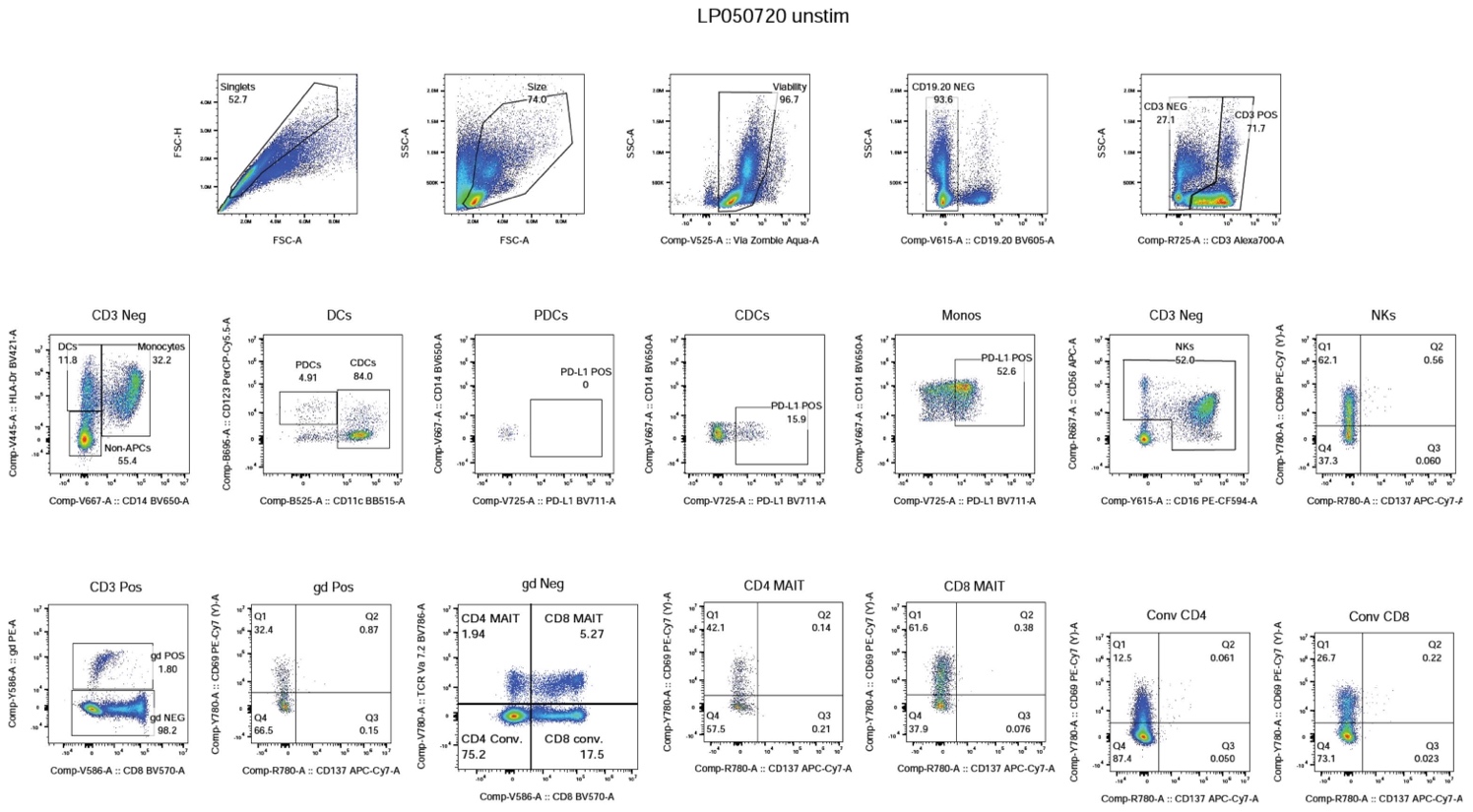


**A**

**B**


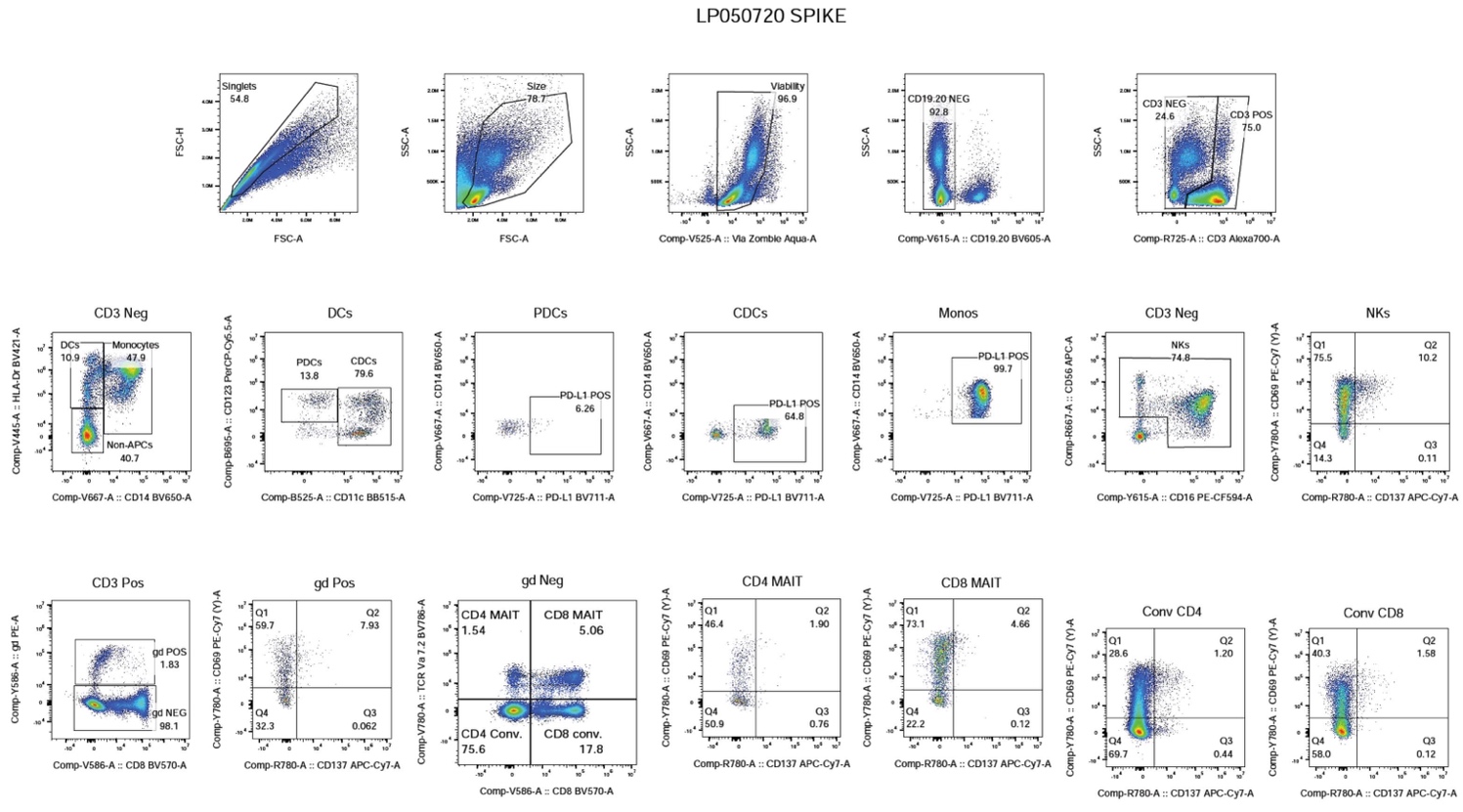


**Figure S1.** **Gating strategy.** **Panel A**: unstimulated PBMC; **Panel B**: Spike Megapool-stimulated PBMC.

B

**Figure S2. Comparison of antibody concentrations in children with or without prior SARS-CoV-2 infection**. Data were derived from 29 children at enrollment in the COVID-19 vaccine trial (pre-vaccination). Antibodies were measured by multiplex microsphere immunoassay. Graphs show mean fluorescence intensity of each participant for receptor binding domain (RBD) and nucleocapsid (N), medians and 25^th^ and 75^th^ quartiles. p values were calculated with Mann-Whitney test.

**Figure S3. Adaptive and innate immune responses to a COVID-19 mRNA vaccine in children with previous SARS CoV-2 infection.** Data were derived from 5 SARS-CoV-2-infected children with paired PBMC samples before and after vaccination. Graphs show paired proportions of the activated innate immune cells indicated on the figure before and after stimulation in Spike (S) peptide-stimulated PBMC after subtraction of unstimulated controls (S-medium). Activated CD4+ and CD8+ Tconv, NK and γδ T cells were identified by the expression of CD69 and CD137 markers and measured out of total CD4+ and CD8+ Tconv, NK and γδ T cells, respectively. Activated monocytes and cDC were identified by the expression of PDL-1 and were expressed as proportions of the total monocytes and cDC, respectively. p values were calculated with Wilcoxon matched-pairs signed rank test.

**Figure S4. Conventional CD8+ T cell responses to the first dose of COVID-19 mRNA vaccine in children without prior SARS CoV-2 infection**. Data were derived from 9 children with paired samples available for testing. Graphs show proportions of CD8+CD69+CD137+ T cells in Spike (S) peptide-stimulated PBMC after subtraction of unstimulated controls (S-medium) out of total CD8+ T cells. p value shown on the graph was calculated by Wilcoxon matched-pairs signed rank test.
